# AVR: Abstractly Verifying Reachability

**DOI:** 10.1007/978-3-030-45190-5_23

**Published:** 2020-03-13

**Authors:** Aman Goel, Karem Sakallah

**Affiliations:** 8 Johannes Kepler University, Linz, Austria; 9 University of Birmingham, Birmingham, UK; University of Michigan, Ann Arbor, MI 48105 USA

## Abstract

We present AVR, a push-button model checker for verifying state transition systems directly at the source-code level. AVR uses information embedded in the word-level syntax of the design representation to automatically perform scalable model checking by combining a novel syntax-guided abstraction-refinement technique with a word-level implementation of the IC3 algorithm. AVR provides independently-verifiable certificates that offer provable assurance and are easy to relate to the word-level system. Moreover, proof certificates can be further used in innovative ways to extract key design information and are useful in a growing number of applications.

### 1 Introduction

Model checking [27,28] techniques based on incremental induction (like IC3 [19,31]) have gained significant success [21] due to their property-directed nature and clever use of incremental SAT solving. Bit-level implementations of IC3, however, struggle with scalability due to being overwhelmed by low-level propositional learning [33]. Rapid advances in SMT solving [54,12] offer a solution and allow for performing IC3 directly at the word level by combining the incremental induction algorithm with an abstraction-refinement procedure [18,41,23,34].

AVR [2] is a model checker designed, primarily, for verifying *safety* properties of hardware. It uses *syntax-guided abstraction* [34], a generalization of implicit predicate abstraction [22], to perform IC3-style reachability on a *first-order logic* encoding of the transition relation resulting in word-level clause learning. Upon termination, AVR will either produce a *proof certificate*, in the form of a state formula representing an *inductive invariant*, if the safety property holds or a counterexample execution trace if it fails. In both cases, confidence in the verification output is achieved by using an external proof checker to independently confirm the correctness of the proof certificate or a trace simulator depicting the sequence of transitions leading to the failure. Beyond hardware, these features allow AVR to be used in innovative ways including the verification of distributed protocols defined over unbounded domains [44,45]. AVR also provides a variety of complementary verification techniques, such as data abstraction and interpolation, to increase its scalability, as well as useful utilities, such as design statistics and graphical visualizations, to provide high-level insights on the input design. AVR was independently evaluated to be the best word-level verifier in the single bit-vector track of Hardware Model Checking Competition (HWMCC) 2019 [17].

### 2 Motivation

Consider a predicate $p := (a + b < 1)$ defined over two 32-bit variables $a$ and $b$. An equivalent propositional-level representation of $p$ will involve a bit-blasted expression involving 64 Boolean variables and several hundred clauses. As a consequence, bit-level model checking algorithms do not scale as variable bit widths increase and suffer from the so-called *state-space explosion* problem [26].

AVR derives its motivation from the fact that the word-level representation of a problem contains useful high-level information that can be exploited for better scalability. Building on our previous work [33,34], AVR uses this insight to infer an implicit syntax-guided abstraction using terms built from objects present in the word-level syntactic description of the problem (like $a$, $b$, 1, $+$, $<$). The approach can be further combined with data abstraction using uninterpreted functions [20,11] to simplify reasoning for the underlying query solver. This, coupled with efficient SMT solving, allows for an effective word-level model checking algorithm that can scale better than bit-level engines for a variety of verification problems. Moreover, the underlying induction-based verification procedure has the unique strength of producing word-level proof certificates that are useful in a variety of applications [32,37,45,44].

### 3 System Architecture

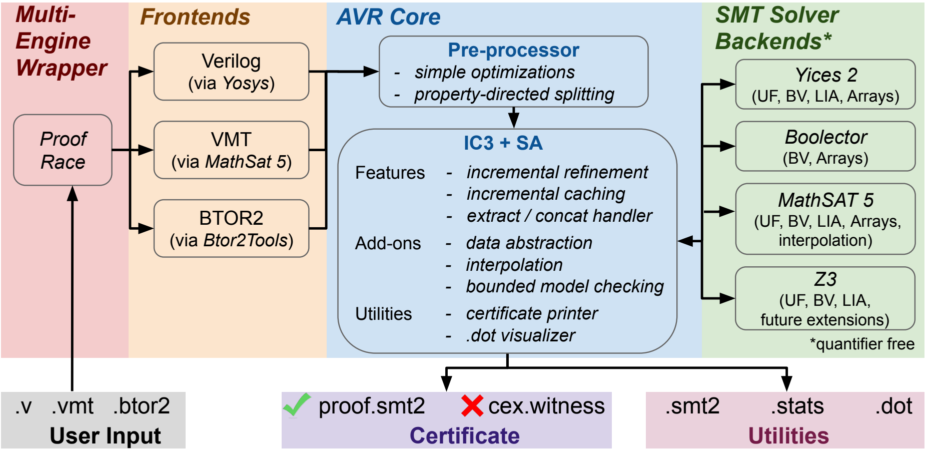

**Fig. 1:** Verification flow with AVR
UF: uninterpreted functions, BV: bit-vectors, LIA: linear integer arithmetic

Fig. 1 shows the architecture and verification flow of AVR.

*Frontends* in AVR extract the model checking problem from inputs in different formats using openly-available tools.

- Verilog + SystemVerilog Assertions [9] (using Yosys [55])
- VMT [8] (using MathSAT 5 [24])
- BTOR2 [51] (using Btor2Tools [3])

*AVR core* performs IC3 with syntax-guided abstraction (IC3+SA) and implements several verification techniques and utilities (detailed in §3.1, §3.2).

*SMT solver backends* use the latest versions of state-of-the-art SMT solvers (Yices 2 [30], Boolector [50], MathSAT 5 [24] and Z3 [48]) to efficiently integrate incremental solver reasoning with *AVR core* using a C++ interface.

*Multi-engine wrapper* allows for process-level parallelism by running multiple instances of AVR in parallel using *proof race* (as elaborated later in §3.3).

#### 3.1 Techniques

At its core, AVR implements a word-level IC3 procedure where terms in the implicit syntax of the problem are used as building blocks to perform IC3-style clause learning at the word level using SMT solving. The key differences between IC3+SA [34], as implemented in AVR, and bit-level IC3 [19,31] can be summarized as follows:

- IC3+SA uses relations defined over syntax terms (referred as *atoms*) instead of individual state bits to implicitly represent an abstract state space.
- SMT solving is used instead of propositional SAT solving for solver reasoning.
- Counterexample-guided abstraction refinement [25] is used to automatically eliminate the spurious behavior in the syntactically abstracted domain by identifying new terms from the proof of unsatisfiability [42].

Within the core IC3+SA framework, AVR implements several optimizations and important features that are helpful in improving model checking performance.

**Core features**

- *Pre-processor optimizations* perform simple transformations to standardize and optimize the input model extracted from different input formats.
- *Incremental refinement* performs abstract counterexample analysis in an incremental fashion by using single-step solver queries instead of conventional multi-step path queries.
- *Incremental caching* allows caching frequently-used data structures to speed up incremental SMT solving (at the cost of increasing memory usage).
- *Multiple SMT backends* allow configuring usage of different SMT solvers for different kinds of SMT queries based on the type of query.

**Add-on techniques**

- *Property-directed splitting* breaks wide words at bit-field extraction and concatenation boundaries [10] in a property-directed manner.
- *Data abstraction* focuses on the control structure of the problem by combining IC3+SA with data abstraction which converts data operations to uninterpreted functions [20,11,41],.
- *Interpolation* adds Craig interpolants [46] and incremental refinement to extract new terms from a spurious abstract counterexample.
- *Extract/Concat handler* adds a *novel* dedicated engine to deal with lightweight interpretation of bit-field extraction and concatenation operations.

- *Bounded model checking* (BMC) [15] allows for an alternative to the IC3+SA engine for quick bug hunting, especially for shallow bugs.
- *Other options* include adding global assumptions *lazily*, minimizing proof certificates, making syntax-guided abstraction closer to (resp. farther from) implicit predicate abstraction by decreasing (resp. increasing) abstraction *granularity*, exploiting *randomness* during solving, and a few others.

**Utilities**

AVR also provides a number of useful utilities to the user including:
- Printing the problem in SMT-LIB format [13].
- Graphical visualizations of the problem and the word-level clause learning.
- Detailed statistics report on the input design and the verification run.

#### 3.2 Certificates

Once a model checking problem is solved, there can be two possible outcomes: either the property holds (safe), or it fails (unsafe).

If the property holds, IC3+SA produces an inductive invariant, i.e. an approximate fixpoint that establishes the property to be true in *all* executions of the system. Inductive invariants act as proof certificates that guarantee the correctness of the verification outcome. AVR prints such proof certificates directly in the SMT-LIB format, which allows for independent checking of their correctness using an external SMT solver like Yices 2 or Z3. Since proof certificates are in the word-level format, they are human-readable and much easier to relate to the word-level input directly at the source-code level (as against bit-level invariants which are usually too hard to understand). Proof certificates have many useful applications, including the derivation of inductive validity cores [32], gaining deeper insights on design behavior, deriving assume-guarantee verification conditions [37,53], deriving helper assertions during multi-property verification [36,29], and generalizing to quantified domains (as elaborated later in §4.3).

When the property fails, AVR produces a counterexample trace that establishes how to reach a bad state (a state where the property is false) starting from an initial state. AVR prints the counterexample witness in BTOR2 witness format [51], which allows for independent verification of the execution trace using a BTOR2 witness simulator [4]. This allows the designer to debug and pin-point the source of error by analyzing the execution leading to the buggy state.

#### 3.3 Proof Race

AVR supports a variety of configurations and add-on features (as discussed in §3.1). Without detailed knowledge of the input, it is hard to tell upfront which technique will perform the best. Different configurations are useful to tackle different types of problems, though manually trying different configurations can become tedious for the user. To counter this, AVR offers a multi-engine wrapper called *proof race* that automatically runs multiple instances of AVR with different configurations in parallel and offers process-level parallelism. Given a set of specified resource limits, proof race initiates multiple AVR instances and terminates execution as soon as one of these instances successfully *races* to the result. Such a portfolio-based approach is crucial in practice for fast verification performance since no single technique performs best in all cases [21,16]. It is also further strengthened by complementing AVR's word-level techniques with state-of-the-art model checking engines like ABC *dprove* [14], IC3ia [23] etc.

## 4 Case Studies[1]

### 4.1 Apache Buffer Overflow

We consider patched versions of two buffer overflow vulnerabilities [40] from standard modules of the Apache web server [1].

*apache-escape-absolute* corrects a high severity vulnerability `CVE2006-3747` [7] that fixes the out-of-bounds buffer overflow exploitation which allows a remote attacker to cause a denial of service and execute arbitrary code via crafted URLs. The patched version corrects a check ($c <$ `TOKEN_SZ`) to ($c <$ `TOKEN_SZ` $- 1$).

*apache-get-tag* fixes a medium severity vulnerability `CVE-2004-0940` [6] that exploits a buffer overflow when copying user-supplied tag strings into finite buffers. A local attacker may leverage this issue to execute arbitrary code on the affected computer with the privileges of the affected Apache server. The patched version corrects a check that validates the length of the tag strings.

In less than a minute, AVR successfully verifies that both of these buffer overflow exploits are unreachable in the patched versions for *any* buffer size. AVR also provides human-readable proof certificates that are externally verified using Z3, and provides provable assurance against these security vulnerabilities.

### 4.2 Public Key Authentication Protocol

The Needham-Schroeder public key authentication protocol [49] allows establishing mutual authentication between an initiator $A$ and a responder $B$, after which some session involving the exchange of messages between them can take place. Unfortunately, this protocol is vulnerable to a man-in-the-middle attack [43]. If an intruder $I$ can persuade $A$ to initiate a session with him, he can relay the messages to $B$ and convince $B$ that he is communicating with $A$.

We consider an instance of the protocol from HWMCC'19 [17,52] with 3 initiators and responders each, and with an unsafe state defined as a responder being finished authentication with the intruder as a party. Within a minute, AVR finds an execution trace that establishes how to reach an unsafe state. The counterexample witness produced by AVR can be replayed using the BtorSIM simulator [4] to verify the execution trace and to debug the protocol.

### 4.3 Verifying Distributed Protocols

Beyond verifying model checking problems from finite domains, AVR has shown preliminary application in the verification of distributed protocols, which are

[1] All results presented in this paper can be replicated from [35,5].

generally expressed over unbounded domains (with an unbounded number of clients, servers, epochs, messages, etc.). The I4 system [45,44] demonstrates how AVR can be used to verify a simpler finite version of the protocol, followed by generalizing AVR's proof certificates to the unbounded domain. For example, a finite-domain invariant saying "clients $C_1$ and $C_2$ cannot both link to the server $S$" i.e. $\neg(link(C_1, S) \wedge link(C_2, S))$ can be generalized to the unbounded domain as "no two different clients can both link to a server" i.e. $\forall_{C_1,C_2,S}\ (C_1 \neq C_2) \implies \neg(link(C_1, S) \wedge link(C_2, S))$.

### 5 Strengths

*Control-centric* properties, where much of the complexity lies in the control logic (such as sequential equivalence checking, microprocessor instruction control unit, key-value store) are much easier to verify using AVR. Syntax-guided abstraction hides the domain complexity outside of the problem syntax, and automatically separates important control-flow details from the irrelevant data component. This, combined with data abstraction, allows for scalable model checking with the capacity to scale independently of the variable bit widths [33,34].

*Push-button verification* using AVR eliminates the need for tedious human intervention in verification (such as manual identification of abstraction predicates, manually adding helper assertions) by automatic incremental construction of abstraction and word-level clauses using the IC3+SA algorithm.

*Provable assurance* on the verification outcome is guaranteed by AVR using independently-checkable proof certificates and counterexample traces.

*Useful utilities* that AVR provides, such as support for multiple input formats, efficient integration with state-of-the-art SMT solvers, proof race, high-level system statistics, graphical visualizations, etc. contribute to a user-friendly experience and ease of use.

### 6 Limitations

*Heavy data dependency* can make word-level techniques in AVR ineffective for certain problems, especially when a majority of bit-precise values in the data domain play an important role (for example, puzzle solving problems like Tower of Hanoi [39], Peg Solitaire [38], etc. formulated as reachability problems [52]). Logic synthesis and bit-level optimizations [14,47] can be very useful for such problems and help bit-level checkers perform better than word-level techniques by significantly decreasing the problem complexity at the bit level.

*First-order logic fragments* beyond quantifier-free bit-vectors, arrays and uninterpreted functions (such as non-linear arithmetic, floating-point numbers, quantifiers, etc.) and properties beyond safety (such as *liveness* and *fairness*) have limited support in the current tool implementation. AVR's primary focus has been on verification of safety properties defined on hardware systems.

### 7 Conclusions

AVR provides a variety of techniques to efficiently perform automatic word-level verification using SMT solvers with provable guarantees and security. AVR has been effective in hardware verification [17,33,34] and shows significant promise for the verification of distributed protocols [44,45]. In the future, we plan to address some of its current limitations and extend its application to practical verification problems beyond the hardware domain.

Data Availability Statement and Acknowledgments. The software and datasets generated and analyzed during the current study are available in the Zenodo repository: https://doi.org/10.5281/zenodo.3677545. The authors would like to thank Ranan Fraer, Ravi Prakash, Habeeb Farah and Ziyad Hanna from Cadence Design Systems for their help in shaping some of the concepts presented in this paper.
